# Joint *L*_1/2_-Norm Constraint and Graph-Laplacian PCA Method for Feature Extraction

**DOI:** 10.1155/2017/5073427

**Published:** 2017-04-02

**Authors:** Chun-Mei Feng, Ying-Lian Gao, Jin-Xing Liu, Juan Wang, Dong-Qin Wang, Chang-Gang Wen

**Affiliations:** ^1^School of Information Science and Engineering, Qufu Normal University, Rizhao 276826, China; ^2^Library of Qufu Normal University, Qufu Normal University, Rizhao 276826, China

## Abstract

Principal Component Analysis (PCA) as a tool for dimensionality reduction is widely used in many areas. In the area of bioinformatics, each involved variable corresponds to a specific gene. In order to improve the robustness of PCA-based method, this paper proposes a novel graph-Laplacian PCA algorithm by adopting *L*_1/2_ constraint (*L*_1/2_ gLPCA) on error function for feature (gene) extraction. The error function based on *L*_1/2_-norm helps to reduce the influence of outliers and noise. Augmented Lagrange Multipliers (ALM) method is applied to solve the subproblem. This method gets better results in feature extraction than other state-of-the-art PCA-based methods. Extensive experimental results on simulation data and gene expression data sets demonstrate that our method can get higher identification accuracies than others.

## 1. Introduction

With the rapid development of gene-chip and deep-sequencing technologies, a lot of gene expression data have been generated. It is possible for biologists to monitor the expression of thousands of genes with the maturation of the sequencing technology [1–3]. It is reported that a growing body of research has been used to select the feature genes from gene expression data [4–6]. Feature extraction is a typical application of gene expression data. Cancer has become a threat to human health. Modern medicine has proved all cancers are directly or indirectly related to genes. How to identify what is believed to be related to cancer has become a hotspot in the field of bioinformatics. The major bottleneck of the development of bioinformatics is how to build an effective approach to integrate and analyze the expression data [7].

One striking feature of gene expression data is the case that the number of genes is far greater than the number of samples, commonly called the high-dimension-small-sample-size problem [8]. Typically this means that expression data are always with more than thousands of genes, while the size of samples is generally less than 100. The huge expression data make them hard to analyze, but only a small size of genes can control the gene expression. More attention has been attached to the importance of feature genes by modern biologists. Correspondingly, it is especially important how to discover these genes effectively, so many dimensionality reduction approaches are proposed.

Traditional dimensionality reduction methods have been widely used. For example, Principal Component Analysis (PCA) recombines the original data which have a certain relevance into a new set of independent indicators [9–11]. However, because of the sparsity of gene regulation, the weaknesses of traditional approaches in the field of feature extraction become increasingly evident [12, 13]. With the development of deep-sequencing technique, the inadequacy of conventional methods is emerging. Within the process of feature selection on biological data, the principal components of PCA are dense, which makes it difficult to give an objective and reasonable explanation on the significance of biology. PCA-based methods have achieved good results in the application of feature extraction [3, 12]. Although this method shows the significance of sparsity in the aspect of handling high dimensional data, there are still a lot of shortcomings in the algorithm.

1. The high dimensionality of data poses a great challenge to the research, which is called data disaster.
2. Facing with millions of data points, it is reasonable to consider the internal geometric structure of the data.
3. Gene expression data usually contain a lot of outliers and noise, but the above methods cannot effectively deal with these problems.

With the development of graph theory [14] and manifold learning theory [15], the embedded structure problem has been effectively resolved. Laplacian embedding as a classical method of manifold learning has been used in machine learning and pattern recognition, whose essential idea is recovery of low dimensional manifold structure from high dimensional sampled data. The performance of feature extraction will be improved remarkably after joining Laplacian in gene expression data. In the case of maintaining the local adjacency relationship of the graph, the graph can be drawn from the high dimensional space to a low dimensional space (drawing graph). However, graph-Laplacian cannot dispose outliers.

In the field of dimensionality reduction, *L*_*p*_  (0 < *p* < 1)-norm was getting more and more popular to replace *L*_1_, which was first proposed by Nie et al. [16]. Research shows that a proper value of *p* can achieve a more exact result for dimensionality reduction [17]. Furthermore, Xu et al. developed an simple iterative thresholding representation theory for *L*_1/2_-norm [18], which was similar to the notable iterative soft thresholding algorithm for the solution of *L*_0_ [19] and *L*_1_-norm [20]. Xu et al. have shown that *L*_*p*_-norm generates more better solution than *L*_1_-norm [21]. Besides, among all regularization with *p* in 0,1/2], there is no obvious difference. However, when *p* ∈ [1/2,1, the smaller *p* is, the more effective result will be [17]. This provides a motivation to introduce *L*_1/2_-norm constraint into original method. Since the error of each data point is calculated in the form of the square. It will also cause a lot of errors while the data contains some tiny abnormal values.

In order to solve the above problems, we propose a novel method based on *L*_1/2_-norm constraint, graph-Laplacian PCA (*L*_1/2_ gLPCA) which provides a good performance. In summary, the main work of this paper is as follows. (1) The error function based on *L*_1/2_-norm is used to reduce the influence of outliers and noise. (2) Graph-Laplacian is introduced to recover low dimensional manifold structure from high dimensional sampled data.

The remainder of the paper is organized as follows.  Section 2 provides some related work. We present our formulation and algorithm for *L*_1/2_-norm constraint graph-Laplacian PCA in Section 3. We evaluate our algorithm on both simulation data and real gene expression data in Section 4. The correlations between the identified genes and cancer data are also included. The paper is concluded in Section 5.

## 2. Related Work

### 2.1. Principal Component Analysis

In the field of bioinformatics, the principal components (PCs) of PCA are used to select feature genes. Assume **X** = (**x**_1_,…, **x**_*n*_) ∈ **R**^*m*×*n*^ is the input data matrix, which contains the collection of *n* data column vectors and *m* dimension space. Traditional PCA approaches recombine the original data which have a certain relevance into a new set of independent indicators [9]. More specifically, this method reduces the input data to *k*-dim (*k* < *n*) subspace by minimizing:

(1) minU,V X−UVTF2s.t. VTV=I,

where each column of **U** = (**u**_1_,…, **u**_*k*_) ∈ **R**^*m*×*k*^ is the principal directions and **V**^*T*^ = (**v**_1_,…, **v**_*n*_) ∈ **R**^*k*×*n*^ is the projected data points in the new subspace.

### 2.2. Graph-Laplacian PCA

Since the traditional PCA has not taken into account the intrinsic geometrical structure within input data, the mutual influences among data may be missed during a research project [9]. With the increasing popularity of the manifold learning theory, people are becoming aware that the intrinsic geometrical structure is essential for modeling input data [15]. It is a well-known fact that graph-Laplacian is the fastest approach in the manifold learning method [14]. The essential idea of graph-Laplacian is to recover low dimensional manifold structure from high dimensional sampled data. PCA closely relates to *K*-means clustering [22]. The principal components *V* are also the continuous solution of the cluster indicators in the *K*-means clustering method. Thus, it provides a motivation to embed Laplacian to PCA whose primary purpose is clustering [23, 24]. Let symmetric weight matrix **W** ∈ **R**^*n*×*n*^ be the nearest neighbor graph where **W**_*ij*_ is the weight of the edge connecting vertices *i* and *j*. The value of **W**_*ij*_ is set as follows:

(2) $$\begin{array}{c} W_{ij}=\left\{\begin{array}{cc} 1 & \text{if }x_{i}\in N_{k}\left(x_{j}\right)\text{ or }x_{j}\in N_{k}\left(x_{i}\right),\\ 0 & \text{otherwise}, \end{array}\right. \end{array}$$

where **N**_*k*_(**x**_*i*_) is the set of *k* nearest neighbors of **x**_*i*_. **V**^*T*^ = (**v**_1_,…, **v**_*n*_) ∈ *R*^*k*×*n*^ is supposed as the embedding coordinates of the data and **D** = diag (**d**_1_,…, **d**_*n*_) is defined as a diagonal matrix and **d**_*i*_ = ∑_*j*_**W**_*ij*_. **V** can be obtained by minimizing:

(3) minV ∑i,j=1nvi−vj2Wij=trVTD−WV=trVTLVs.t. VTV=I,

where **d**_*i*_ is the column or row sums of **W** and **L** = **D** − **W** is named as Laplacian matrix. Simply put, in the case of maintaining the local adjacency relationship of the graph, the graph can be drawn from the high dimensional space to a low dimensional space (drawing graph). In the view of the function of graph-Laplacian, Jiang et al. proposed a model named graph-Laplacian PCA (gLPCA), which incorporates graph structure encoded in **W** [23]. This model can be considered as follows:

(4) minU,V J=X−UVTF2+α trVTLVs.t. VTV=I,

where *α* ≥ 0 is a parameter adjusting the contribution of the two parts. This model has three aspects. (a) It is a data representation, where **X**≃**UV**^*T*^. (b) It uses **V** to embed manifold learning. (c) This model is a nonconvex problem but has a closed-form solution and can be efficient to work out.

In (4), from the perspective of data point, it can be rewritten as follows:

(5) minU,V J=∑j=1nXn−UvnTF2+α trvnTLvns.t. VTV=I.

In this formula, the error of each data point is calculated in the form of the square. It will also cause a lot of errors while the data contains some tiny abnormal values. Thus, the author formulates a robust version using *L*_2,1_-norm as follows:

(6) minU,V X−UVT2,1+α trVTLVs.t. VTV=I,

but the major contribution of *L*_2,1_-norm is to generate sparse on rows, in which the effect is not so obvious [3, 25].

## 3. Proposed Algorithm

Research shows that a proper value of *p* can achieve a more exact result for dimensionality reduction [17]. When *p* ∈ [1/2,1, the smaller *p* is, the more effective result will be [17]. Then, Xu et al. developed a simple iterative thresholding representation theory for *L*_1/2_-norm and obtained the desired results [18]. Thus, motivated by former theory, it is reasonable and necessary to introduce *L*_1/2_-norm on error function to reduce the impact of outliers on the data. Based on the half thresholding theory, we propose a novel method using *L*_1/2_-norm on error function by minimizing the following problem:

(7) minU,V X−UVT1/21/2+α trVTLVs.t. VTV=I,

where *L*_1/2_-norm is defined as ‖**A**‖_1/2_^1/2^ = ∑_*j*_^*n*^∑_*j*_^*m*^|**a**_*ij*_|^1/2^, **X** = (**x**_1_,…, **x**_*n*_) ∈ **R**^*m*×*n*^ is the input data matrix, and **U** = (**u**_1_,…, **u**_*k*_) ∈ **R**^*m*×*k*^ and **V**^*T*^ = (**v**_1_,…, **v**_*n*_) ∈ **R**^*k*×*n*^ are the principal directions and the subspace of projected data, respectively. We call this model graph-Laplacian PCA based on *L*_1/2_-norm constraint (*L*_1/2_ gLPCA).

At first, the subproblems are solved by using the Augmented Lagrange Multipliers (ALM) method. Then, an efficient updating algorithm is presented to solve this optimization problem.

### 3.1. Solving the Subproblems

ALM is used to solve the subproblem. Firstly, an auxiliary variable is introduced to rewrite the formulation (4) as follows:

(8) minU,V,S S1/21/2+α tr VTD−WV,s.t. S=X−UVT,  VTV=I.

The augmented Lagrangian function of (8) is defined as follows:

(9) LμS,U,V,Λ=S1/21/2+tr ΛTS−X+UVT+μ2S−X+UVTF2+α trVTLV,s.t. VTV=I,

where Λ is Lagrangian multipliers and *μ* is the step size of update. By mathematical deduction, the function of (9) can be rewritten as

(10) LμS,U,V,Λ=S1/21/2+μ2S−X+UVT+ΛμF2+α trVTLV,s.t. VTV=I.

The general approach of (10) consists of the following iterations:

(11) Sk+1=arg minS LμS,Uk,Vk,Λk,Vk+1=v1,…,vk,Uk+1=MVk,Λk+1=Λk+μSk+1−X+UkVTk,μk+1=ρμk.

Then, the details to update each variable in (11) are given as follows.

*Updating* **S**. At first, we solve **S** while fixing **U** and **V**. The update of **S** relates the following issue:

(12) $$\begin{array}{c} S^{k+1}=arg\;\underset{S}{min}{\left\|\;S\;\right\|}_{1/2}^{1/2}+\frac{\mu }{2}{\left\|\;S-X+U^{k}{V^{T}}^{k}+\frac{\Lambda^{k}}{\mu }\;\right\|}_{F}^{2}, \end{array}$$

which is the proximal operator of *L*_1/2_-norm. Since this formulation is a nonconvex, nonsmooth, non-Lipschitz, and complex optimization problem; an iterative half thresholding approach is used for fast solution of *L*_1/2_-norm and summarizes according to the following lemma [18].

**Lemma 1 .**

The proximal operator of *L*_1/2_-norm minimizes the following problem:

(13) $$\begin{array}{c} \underset{X\in R^{m\times n}}{min}{\left\|\;X-A\;\right\|}_{F}^{2}+\lambda {\left\|\;X\;\right\|}_{1/2}^{1/2}, \end{array}$$

which is given by

(14) $$\begin{array}{c} X^{*}=H_{\lambda }\left(\;A\;\right)=U\mathrm{diag}\left(\;H_{\lambda }\left(\;\sigma \;\right)\;\right)V^{T}, \end{array}$$

where **H**_*λ*_(*σ*)≔(*h*_*λ*_(*σ*_1_), *h*_*λ*_(*σ*_2_),…, *h*_*λ*_(*σ*_*n*_))  ^*T*^ and  *h*_*λ*_(*σ*_*i*_) is the half threshold operator and defined as follows:

(15) hλσi=23σi1+cos2π3−23ψλσi,if  σi>5434λ2/30,otherwise,

where *ψλ*(*σ*_*i*_) = arccos⁡((*λ*/8)(|*σ*_*i*_|/3)^−2/3^).

*Solving* **U** *and* **V**. Here, we solve **U** while fixing others. The update of **U** amounts to solving

(16) $$\begin{array}{c} U^{k+1}=arg\;\underset{U}{min}\frac{\mu }{2}{\left\|\;S^{k}-X+U^{k}V^{T^{k}}+\frac{\Lambda^{k}}{\mu }\;\right\|}_{F}^{2}. \end{array}$$

Letting **X** − **S** − Λ/*μ* = **M**, (16) becomes **U**^*k*+1^ = arg min_**U**_(*μ*/2)‖**M** − **UV**^*T*^*k*^^‖_*F*_^2^, taking partial derivatives of **U** as follows:

(17) $$\begin{array}{c} \frac{\partial J}{\partial U}=-\mu \left(\;M-UV^{T^{k}}\;\right)V. \end{array}$$

Setting the partial derivatives to 0, we have

(18) $$\begin{array}{c} U^{k+1}=MV^{k}. \end{array}$$

Then, we solve **V** while fixing others. Similarly, letting **X** − **S** − Λ/*μ* = **M**, **U** = **MV**, the update of **V** can be listed as follows:

(19) Vk+1=arg minVμ2M−MVVTF2+α trVTLV,s.t. VTV=I.

By some algebra, we have

(20) Vk+1arg minVM−MVVTF2+2αμtrVTLV=arg minV trMMT−2trMMT2+2αμtrVTLV=arg minV tr VT−MTM+2αμLV.

Therefore, (19) can be rewritten as follows:

(21) Vk+1=arg minV tr VT−MTM+2αμLV,s.t. VTV=I.

Thus, the optimal **V**^*k*+1^ can be obtained by calculating eigenvectors

(22) $$\begin{array}{c} V^{k+1}=\left(\;v_{1},\ldots ,v_{k}\;\right), \end{array}$$

which corresponds to the first *k* smallest eigenvalues of the matrix *G*_*α*_ = −**M**^*T*^**M** + 2*α ***L**/*μ*.

*Updating *Λ* and μ*. The update of Λ and *μ* is standard:

(23) Λk+1=Λk+μSk+1−X+UkVTk,μk+1=ρμk,

where *ρ* > 1 is used to update the parameter *μ*. Since the value of *ρ* is usually bigger than 1, and over a large number of experiments, we find *ρ* = 1.1*～*1.5 are good choice. We selected *ρ* = 1.2 in such practice conditions.

The complete procedure is summarized in Algorithm 1.

### 3.2. Properties of Algorithm

We set *ρ* = 1.2 through all our gene expression data experiments. Whereas we introduce *σ*_*m*_, *σ*_*l*_ is the largest eigenvalue of matrix **M**^*T*^**M** and **L** to normalize them, respectively. Setting

(24) $$\begin{array}{c} \frac{2\alpha }{\mu }=\frac{\beta }{1-\beta }\frac{\sigma_{m}}{\sigma_{l}}, \end{array}$$

where *β* is the parameter to substitute for *α*, (20) can be rewritten as

(25) V=arg minV tr VT1−βI−MTMσm+2βμLσlV,s.t. VTV=I.

Therefore, the solution of **V** can be expressed by the eigenvectors of *G*_*β*_:

(26) $$\begin{array}{c} G_{\beta }=\left(\;1-\beta \;\right)\left(\;I-\frac{M^{T}M}{\sigma_{m}}\;\right)+\frac{2\beta }{\mu }\frac{L}{\sigma_{l}}. \end{array}$$

It is easy to see that *β* should be in the range 0 ≤ *β* ≤ 1. Without *L*_1/2_-norm, there will be standard PCA if *β* = 0. Similarly, when *β* = 1, it reduces to Laplacian embedding.

Furthermore, we rewrite the matrix *G*_*β*_ as follows:

(27) $$\begin{array}{c} G_{\beta }=\left(\;1-\beta \;\right)\left(\;I-\frac{M^{T}M}{\sigma_{m}}\;\right)+\frac{2\beta }{\mu }\left(\;\frac{L}{\sigma_{l}}+\frac{ee^{T}}{n}\;\right), \end{array}$$

where **e** = (1 ⋯ 1)^*T*^ is an eigenvector of *G*_*β*_: *G*_*β*_**e** = (1 − *β*)**e**. We have **Me** = 0, because **X** is centered and it is easy to see that **M** = **X** − **S** − Λ/*μ* is centered. *G*_*β*_ is semipositive definite, because *σ*_*m*_ is the biggest eigenvalue of **M**^*T*^**M**; thus **I** − **M**^*T*^**M**/*σ*_*m*_ is semipositive definite. Meanwhile, it is easy to see that **L** is semipositive definite. Since *G*_*β*_ is a symmetric real matrix that eigenvectors are mutually orthogonal, thus **e** is orthogonal to others. Although we apply **ee**^*T*^/*n* in the Laplacian matrix part, the eigenvectors and eigenvalues do not change, which guarantees that the lowest *k* eigenvectors do not include **e**.

## 4. Experiments

In this section, we compare our algorithm with Laplacian embedding (LE) [26], PCA [9], *L*_0_ PCA, *L*_1_ PCA [12], gLPCA, and RgLPCA [23] on simulation data and real gene expression data, respectively, to verify the performance of our algorithm. Among them, PCA and LE are obtained by adjusting the parameters of gLPCA *β* = 0 and *β* = 1, respectively. Since our algorithm is not sensitive to parameter mu in practice. In the first subsection, we provide the source of simulation data and experimental comparison results. The experimental results and the function of selected genes on real gene expression data with different methods are compared in the next two subsections.

### 4.1. Results on Simulation Data

#### 4.1.1. Data Source

Here, we describe a method to produce simulation data. Supposing we generate the data matrix **A** ∈ **R**^*k*×*j*^, where *k* = 2000 and *j* = 10 are the number of genes and samples, respectively, the simulation data are generated as **A** ~ (0, Σ_4_). Let ${\tilde{v}}_{1}～{\tilde{v}}_{4}$ be four 2000-dimensional vectors; for instance, ${\tilde{v}}_{1k}=1$,  *k* = 1,…, 50, and ${\tilde{v}}_{1k}=0$,  *k* = 51,…, 2000;  ${\tilde{v}}_{2k}=1$,  *k* = 51,…, 100, and ${\tilde{v}}_{2k}=0$,  *k* ≠ 51,…, 100;  ${\tilde{v}}_{3k}=1$,  *k* = 101,…, 150, and ${\tilde{v}}_{3k}=0$,  *k* ≠ 101,…, 150;  ${\tilde{v}}_{4k}=1$,  *k* = 151,…, 200, and ${\tilde{v}}_{4k}=1$,  *k* ≠ 151,…, 200. Given a matrix **E** ~ *N*(0,1) as a noise matrix with 2000-dimension and different Signal-to-Noise Ratio (SNR), which is added into $\tilde{v}$, the four eigenvectors of Σ_4_ can be expressed as ${\tilde{v}}_{k}={\tilde{v}}_{k}/\left\|{\tilde{v}}_{k}\right\|$,  *k* = 1,2, 3,4. Let the four eigenvectors dominate; the eigenvalues of **A** can be denoted as *c*_1_ = 400, *c*_2_ = 300, *c*_3_ = 200, *c*_4_ = 100, and *c*_*k*_ = 1 for *k* = 5,…, 2000.

#### 4.1.2. Detailed Results on Simulation Data

**Figure 1:**
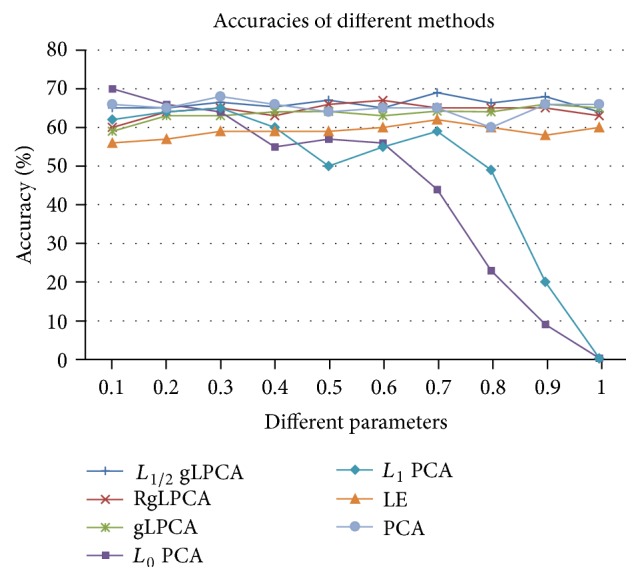
The accuracy of different methods on simulation data with different parameters.

**Figure 2:**
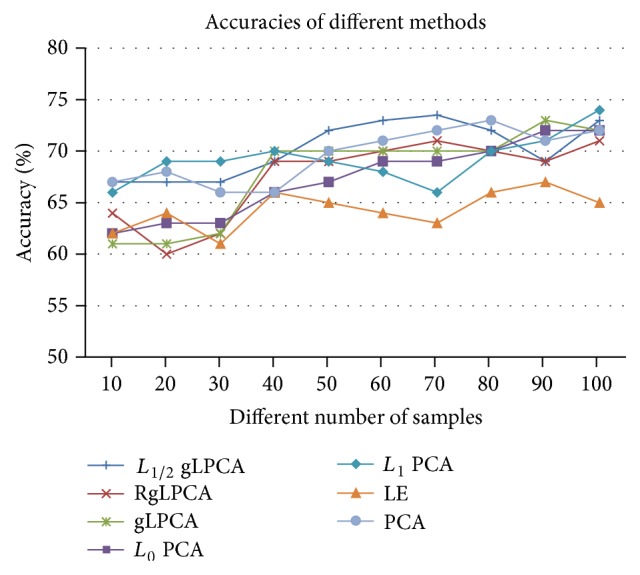
The accuracy of different methods on simulation data with different numbers of samples.

In order to give more accurate experiment results, the average values of the results of 30 times are adopted. For fairness and uniformity, 200 genes are selected by the five methods with their unique parameters. Here, we show the accuracy (%) of these methods. In Figure 1, two factors as two different axes are in the figure. In Figure 2, *x*-axis is the number of samples. *x*-axis is the value of parameter *μ*. The accuracy is defined as follows:

(28) $$\begin{array}{c} \text{Accuracy}=\frac{1}{t}\overset{t}{\underset{i=1}{\sum }}Acc_{i}\times 100\%, \end{array}$$

where *t* is the iterative times and Acc_*i*_ is the identification accuracy of the *i*th time. We define Acc as follows:

(29) $$\begin{array}{c} Acc=\frac{1}{r}\overset{r}{\underset{j=1}{\sum }}\delta \left(\;I_{j},map\left(\;I_{j}\;\right)\;\right), \end{array}$$

where *r* denotes the number of genes, *δ*(*m*, *n*) is a function that equals to 0 if *m* ≠ *n* and equals to 1 if *m* = *n*. We use the function map(*I*) to map the identification of labels. In Figure 1, we show the average accuracies of the seven methods with different sparse parameters while the simulation data is 2000 × 10 and the average accuracy with all parameters is listed in Table 1. In general, if the algorithm is more sensitive to noise and outliers, the deviation will be greater and the accuracy will be greatly reduced. It is worthy to notice that *L*_1/2_ gLPCA works better than other six methods with higher identification accuracies. This means that our algorithm has lower sensitivity to noise and outliers. This table clearly displays the detail of the identification accuracies in different sparse parameters; our method indicates the superiority when the parameter is larger than 0.4 and the curve is more stable. The accuracy of *L*_0_ PCA and *L*_1_ PCA starts a precipitous decline when the parameter is larger than 0.7 and 0.8. Compared with *L*_0_ PCA and *L*_1_ PCA, the methods of *L*_1/2_ gLPCA, RgLPCA, gLPCA, PCA, and LE are not sensitive to the parameter, so there is no substantial change. The stability and average accuracy of various methods can be seen from Table 1.

Furthermore, the number of samples in real gene expression data has a significant influence on the identification accuracy when we select feature gene. Based on this theory, we test different numbers of samples with their best parameters and the average values of the results of 30 times. From the results of Figure 1, we select 0.8 as the parameters of *L*_1/2_ gLPCA, gLPCA, RgLPCA, PCA, and LE. For *L*_0_ PCA and *L*_1_ PCA, we do not change its parameters, since it can obtain the best result from the author's description. The details of average identification accuracies which use seven methods with different sample numbers can be seen from Figure 2. As seen in Figure 2, the accuracy of *L*_1/2_ gLPCA is generally better than other methods and increases with the increase of the number of samples. Besides, Table 2 shows the average accuracy and variance of seven different methods on simulation data with different number of samples. From Table 2, our approach performs better than other methods, even though, in the case of a small number of samples, the accuracy is still high.

### 4.2. Results on Gene Expression Data

In this subsection, the features (genes) are selected by these methods and sent to ToppFun to detect the gene-set enrichment analysis, which is a type of GOTermFinder [27]. The primary role of GOTermFinder is to discover the common of large amounts of gene expression data. The analysis of GOTermFinder provides critical information for the experiment of feature extraction. It is available publicly at https://toppgene.cchmc.org/enrichment.jsp. We set *P* value cutoff to 0.01 through all the experiment. For fair comparison, about *L*_1/2_ gLPCA, RgLPCA, and gLPCA, we both set *β* = 0.5 to control the degree of Laplacian embedding through all experiments in this paper. When *β* = 0, *β* = 1, it results in standard PCA and LE, respectively. Since our algorithm is not sensitive to parameter *μ* mu in practice, we set *μ* = 0.3 through our experiment.

#### 4.2.1. Results on ALLAML Data

The data of ALLAML as a matrix includes 38 samples and 5000 features (genes), which are publicly available at https://sites.google.com/site/feipingnie/file. It is made up of 11 types of acute myelogenous leukemia (AML) and 27 types of acute lymphoblastic leukemia (ALL) [28]. This data contains the difference between AML and ALL, and ALL is divided into T and B cell subtypes. In this experiment, 300 genes are selected and sent to ToppFun. A series of enrichment analyses are conducted on the extracted top 500 genes corresponding to different methods. The complete experimental data have been listed as supplementary data. The *P* value and hit count of top nine terms about molecular function, biological process, and cellular component of ALLAML data by different methods are listed in Table 3. The *P* value is significance for these genes enrichment analysis in these GO terms; the smaller the *P* value is, the more significant these GO terms are. In this Table, the number of hits is the number of genes from input, and the *P* value was influenced by the number of genes from input and so on. Thus, the difference in number of hits is smaller than the difference in *P* value. It shows clearly that our method performs better than compared methods in 8 terms. The lower *P* value shows that the algorithm is less affected by noise and outliers and thus has high efficiency. If the algorithm is affected by noise and outliers significantly, the degree of gene enrichment will be reduced. Nevertheless, LE has the lowest *P* value in term GO: 0098552. From this table, we can see that there are 93 genes in the item of “immune response” which are selected by our method. This item can be considered as the most probable enrichment item, since it has the lowest *P* value. And many researches were focused on the immune status of leukemia [29–32]. Besides, 210 genes associated with leukemia are listed in an article, and 26 out of top 30 genes selected by our method can be found in this article [33]. And 30 genes selected by our method can be found in another published article [34]. The high overlap rate of these genes selected by our method with this published literature approved the effectiveness of our method.

#### 4.2.2. Pathway Search Result on ALLAML Data

**Figure 3:**
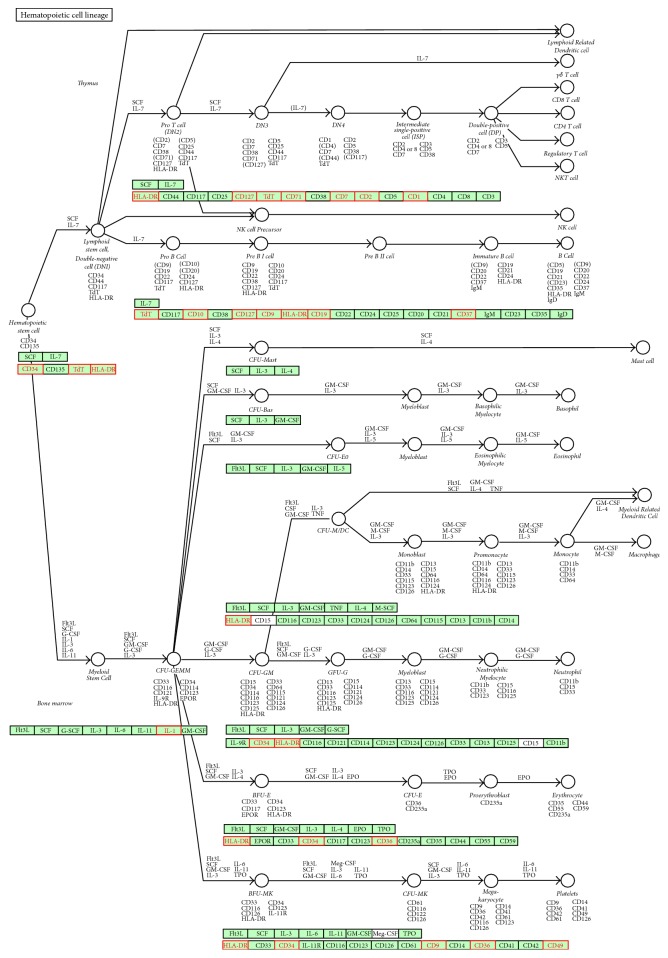
The pathway of hematopoietic cell lineage.

For the sake of the correlations between the selected genes and ALLAML data, the genes selected by *L*_1/2_ gLPCA are proved based on gene-set enrichment analysis (GSEA) that is publicly available at http://software.broadinstitute.org/gsea/msigdb/annotate.jsp. We make analysis by GSEA to compute overlaps for selected genes.  Figure 3 displays the pathway of hematopoietic cell lineage that has highest gene overlaps in this experiment. From Figure 3, 15 genes from our experiment are contained. Among them, HLA-DR occurs seven times. Hematopoietic cell lineage belongs to organismal systems and immune system. On the subject of acute myeloid leukemia (AML), there is consensus about the target cell within the hematopoietic stem cell hierarchy that is sensitive to leukemic transformation, or about the mechanism, that is, basic phenotypic, genotypic, and clinical heterogeneity [35]. Hematopoietic stem cell (HSC) developing from the blood-cell can undergo self-renewal and differentiate into a multilineage committed progenitor cell: one is a common lymphoid progenitor (CLP) and the other is called a common myeloid progenitor (CMP) [36]. A CLP causes the lymphoid lineage of white blood cells or leukocytes, the natural killer (NK) cells and the T and B lymphocytes. A CMP causes the myeloid lineage, which comprises the rest of the leukocytes, the erythrocytes (red blood cells), and the megakaryocytes that produce platelets important in blood clotting. Cells express a stage- and lineage-specific set of surface markers in the differentiation process. So the specific expression pattern of these genes is one way to identify the cellular stages. Related diseases include hemophilia, Bernard-Soulier syndrome, and castleman disease. In medicine, leukemia is a kind of malignant clonal disease of hematopoietic stem cells. Bone marrow transplantation is a magic weapon for the cure of leukemia, by recreating the hematopoietic system to cure leukemia. Generally speaking, when a person has problem in hematopoietic system, it might be related to leukemia [37].

#### 4.2.3. Results on TCGA with PAAD-GE Data

As the largest public database of cancer gene information, The Cancer Genome Atlas (TCGA, https://tcgadata.nci.nih.gov/tcga/) has been producing multimodal genomics, epigenomics, and proteomics data for thousands of tumor samples across over 30 types of cancer. At the same time, as a multidimensional combination of data, five levels of data are involved, such as gene expression (GE), Protein Expression (PE), DNA Methylation (ME), DNA Copy Number (CN), and microRNA Expression (miRExp). Two disease data sets are downloaded from TCGA to be analyzed in the following two experiments. Pancreatic cancer is a type of disease that threatens human health. In this experiment, pancreatic cancer gene expression data (PAAD-GE) is analyzed by these methods. The data of PAAD-GE data as a matrix includes 180 samples and 20502 features (genes). In this subsection, we extract PAAD-GE data to complete this set of comparative experiments and 500 genes are selected and sent to ToppFun. We select top nine terms from molecular function, biological process, and cellular component by *L*_1/2_ gLPCA and compare with other methods. The *P* value and hit count of these terms are listed in Table 4. It is indicated clearly in Table 4 that our method is more stable than other methods, which has lower *P* value in 7 terms. But in terms GO:0045047 and GO:0072599, PCA performs better than other methods. Nevertheless, *L*_1/2_ gLPCA has the same *P* value with gLPCA in terms GO:0045047 and GO:0072599. 196 genes in the item of “extracellular space” are selected by our method.

#### 4.2.4. Pathway Search Result on PAAD-GE Data

**Figure 4:**
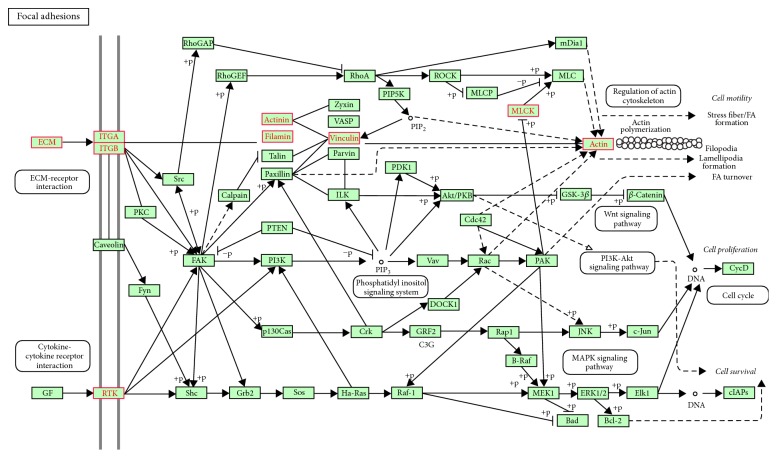
The pathway of focal adhesion.

Similarly as the last experiment, we send our result to GSEA and list the highest genes overlap pathway map in Figure 4. In 1982, Ohhashi reported 4 cases with unique clinical pathological features and is different from normal pancreatic cancer cases, and these 4 cases belong to a completely new clinical type, known as “mucus production type carcinoma (mucin-producing carcinoma, M-pC).” Focal adhesion belongs to cellular processes and cellular community. More specifically, cell-matrix adhesions play important roles in biological processes including cell motility, cell proliferation, cell differentiation, regulation of gene expression, and cell survival. At the cell-extracellular matrix contact points, specialized structures are created and termed focal adhesions, where bundles of actin filaments are fixed to transmembrane receptors of the integrin family through a multimolecular complex of junctional plaque proteins. Integrin signaling is dependent on the nonreceptor tyrosine kinase activities of the FAK and src proteins as well as the adaptor protein functions of FAK, src and Shc to start downstream signaling events. Similar morphological alterations and modulation of gene expression are started by the binding of growth factors to their respective receptors, underling the considerable crosstalk between adhesion- and growth factor-mediated signaling. The early literatures have shown that there is a certain relationship between the pancreatic cancer and focal adhesion [38]. Activation of focal adhesion kinase enhances the adhesion and invasion of pancreatic cancer cells. Besides, Type II diabetes mellitus is another important pathway and is widely believed to be associated with pancreatic cancer; a meta-analysis has examined this association [39].

#### 4.2.5. Correlations between the Selected Genes and PAAD-GE Data

The function of top 7 genes selected by *L*_1/2_ gLPCA is listed in Table 5 based on literatures and GeneCards (http://www.genecards.org/). As can be clearly seen from the table, most of these genetic lesions would likely incur pancreas-related diseases. The etiology of pancreatic cancer is not very clear; it is noted that there is a certain relationship between the incidence of chronic pancreatitis and pancreatic cancer, and we find a significant increase in the proportion of chronic pancreatitis patients with pancreatic cancer. This view is consistent with our experimental result. The clinical observation shows that abdominal pain is the most obvious symptom in the early stage of pancreatic cancer. Some literature on these genes also made a further research as follows. The gene PRSS1 variant likely affects disease susceptibility by altering expression of the primary trypsinogen gene [40]. The pancreatic lipase gene (PNLIP) is located within the genomic region of a bovine marbling quantitative trait locus. PNLIP is a positional and functional candidate for the marbling gene [41].

### 4.3. The Accuracy and Highest Relevance Score

Because ALLAML and PPAD are human disease data sets, we can find them directly from GeneCards and they are publicly available at http://www.genecards.org/.

In order to summarize the experiments on gene expression data, we compute the accuracy and highest relevance score of these methods from GeneCards and list the details in Table 6. The accuracy in Table 6 indicates the proportion of genes which are real associated with the disease in all of the genes selected by these methods. From Table 6, we observe the following. (1) Both PCA and LE commonly provide better accuracy results than *L*_0_ PCA and *L*_1_ PCA, demonstrating the usefulness of PCA and LE. (2) gLPCA has a good performance in some conditions and is unstable. Thus, it is necessary to reduce the effects of outliers and noise. (3) *L*_1/2_ gLPCA and RgLPCA consistently perform better than other methods, but *L*_1/2_ gLPCA has the highest relevance score and highest accuracy.

## 5. Conclusions

This paper investigates a new method of graph-Laplacian PCA (*L*_1/2_ gLPCA) by applying *L*_1/2_-norm constraint on the former method. *L*_1/2_-norm constraint is applied on error function to improve the robustness of the PCA-based method. Augmented Lagrange Multipliers (ALM) method is applied to solve the optimization problem. Extensive experiments on both simulation and real gene expression data have been performed. Results on these two kinds of data show that our proposed method performs better than compared methods. Based on our proposed method, many genes have been extracted to analyze. The identified genes are demonstrated that they are closely related to the corresponding cancer data set.

In future, we will modify the model to improve sparse and robustness of the structure at the same time.

## Supplementary Material

In the supplementary data, "resultALL_AML_data" are the top 500 genes found by our method on the ALLAML dataset, "find_official_name" is the way to find the official name of these genes and "0.5gLPCA" is the experimental code of our method.

## Figures and Tables

**Algorithm 1:**
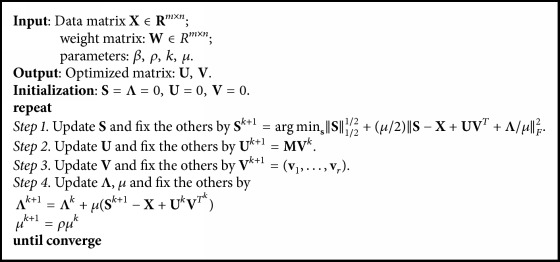
Procedure of *L*_1/2_ gLPCA.

**Table 1:** The average accuracy and variance of different methods on simulation data with different parameters.

Methods	*L*_1/2_ gLPCA	RgLPCA	gLPCA	*L*_0_ PCA	*L*_1_ PCA	PCA	LE
Average accuracy (%)	66.12	65.47	63.53	44.43	48.43	59.00	65.10
Variance	1.48	1.62	1.76	23.60	20.30	1.61	1.97

**Table 2:** The average accuracy and variance of different methods on simulation data with different numbers of samples.

Methods	*L*_1/2_ gLPCA	RgLPCA	gLPCA	*L*_0_ PCA	*L*_1_ PCA	PCA	LE
Average accuracy (%)	70.25	68.25	67.90	67.30	69.20	58.62	69.60
Variance	2.58	3.84	4.41	3.52	2.23	1.79	2.50

**Table 3:** Enrichment analysis of the top 500 genes in the ALLAML data corresponding to different methods.

ID	Name	*L*_1/2_ gLPCA		RgLPCA		gLPCA		*L*_0_ PCA		*L*_1_ PCA		PCA		LE	
		*P*-value	Hit	*P*-value	Hit	*P*-value	Hit	*P*-value	Hit	*P*-value	Hit	*P*-value	Hit	*P*-value	Hit
GO:0006955	Immune response	1.34**E** − 36	93	2.51*E* − 34	91	1.20*E* − 34	91	2.45*E* − 31	87	5.14*E* − 32	89	4.05*E* − 35	91	1.98*E* − 35	91
GO:0002684	Positive regulation of immune system process	2.44**E** − 29	67	2.17*E* − 25	63	1.24*E* − 26	64	3.60*E* − 28	66	1.56*E* − 27	66	8.98*E* − 28	65	3.45*E* − 28	66
GO:0098552	Side of membrane	3.80*E* − 25	46	5.19*E* − 34	45	2.70*E* − 22	43	2.23*E* − 20	41	7.25*E* − 21	42	2.01*E* − 23	44	4.24**E** − 26	47

GO:0009897	External side of plasma membrane	1.83**E** − 17	30	6.34*E* − 16	29	9.51*E* − 14	26	1.14*E* − 13	26	1.41*E* − 12	25	1.31*E* − 16	29	1.83*E* − 14	26
GO:0005615	Extracellular space	2.01**E** − 17	63	8.37*E* − 15	60	2.39*E* − 14	58	6.12*E* − 16	61	3.52*E* − 13	57	2.27*E* − 16	61	4.74*E* − 16	61
GO:0005764	Lysosome	3.49**E** − 17	38	7.43*E* − 16	37	5.46*E* − 14	34	1.20*E* − 14	35	9.22*E* − 11	30	1.08*E* − 15	36	3.49*E* − 16	37

GO:0009986	Cell surface	3.58**E** − 17	48	4.82*E* − 15	45	4.68*E* − 13	42	6.13*E* − 13	42	5.58*E* − 12	41	6.58*E* − 16	46	3.58*E* − 16	46
GO:0042277	Peptide binding	5.03**E** − 14	25	5.92*E* − 13	24	2.85*E* − 11	22	3.33*E* − 11	22	7.54*E* − 08	18	3.09*E* − 12	23	1.80*E* − 10	21
GO:0033218	Amide binding	7.37**E** − 14	26	4.34*E* − 12	24	3.44*E* − 11	23	4.04*E* − 11	23	7.36*E* − 08	19	3.95*E* − 12	24	2.04*E* − 10	22

**Table 4:** Enrichment analysis of the top 500 genes in the PAAD-GE data corresponding to different methods.

ID	Name	*L*_1/2_ gLPCA		RgLPCA		gLPCA		*L*_0_ PCA		*L*_1_ PCA		PCA		LE	
		*P* value	Hit	*P* value	Hit	*P* value	Hit	*P* value	Hit	*P* value	Hit	*P* value	Hit	*P* value	Hit
GO:0005615	Extracellular space	3.20**E** − 93	196	3.56*E* − 80	183	2.18*E* − 72	173	2.742*E* − 61	160	7.82*E* − 61	161	1.44*E* − 58	157	3.20*E* − 89	191
GO:0006614	SRP-dependent cotranslational protein targeting to membrane	2.79**E** − 86	67	6.82*E* − 75	56	1.37*E* − 73	64	3.45*E* − 51	48	8.17*E* − 56	51	7.45*E* − 82	63	2.76*E* − 56	51
GO:0070972	Protein localization to endoplasmic reticulum	1.01**E** − 83	73	2.42*E* − 72	69	6.37*E* − 71	68	5.31*E* − 48	51	4.63*E* − 52	54	2.88*E* − 76	71	4.70*E* − 51	53

GO:0006613	Cotranslational protein targeting to membrane	1.86**E** − 82	67	3.48*E* − 79	65	7.58*E* − 73	64	3.27*E* − 49	48	1.19*E* − 53	51	2.04*E* − 80	66	4.04*E* − 54	51
GO:0045047	Protein targeting to ER	5.01**E** − 82	67	5.19*E* − 74	65	2.00*E* − 71	64	6.04*E* − 49	48	2.33*E* − 53	51	5.80**E** − 82	67	7.90*E* − 54	51
GO:0022626	Cytosolic ribosome	1.34**E** − 81	68	2.13*E* − 75	64	1.44*E* − 70	62	8.30*E* − 44	47	8.45*E* − 48	50	6.34*E* − 74	66	4.01*E* − 51	52

GO:0072599	Establishment of protein localization to endoplasmic reticulum	2.77**E** − 80	67	8.15*E* − 78	66	4.44*E* − 70	64	6.44*E* − 48	48	3.09*E* − 52	51	3.20**E** − 80	67	1.05*E* − 52	51
GO:0005198	Structural molecule activity	1.82**E** − 68	126	2.46*E* − 65	124	6.14*E* − 63	121	3.62*E* − 52	110	5.16*E* − 54	113	3.32*E* − 65	124	1.03*E* − 54	113
GO:0044391	Ribosomal subunit	5.14**E** − 64	69	5.18*E* − 60	65	3.22*E* − 56	63	2.86*E* − 37	49	1.42*E* − 40	52	1.58*E* − 63	68	3.70*E* − 42	53

**Table 5:** The function of top 7 extraction genes.

Gene ID	Gene name	Related GO annotations	Related diseases	Paralogous genes
5644	PRSS1	Serine-type endopeptidase activity	Trypsinogen deficiency and prss1-related hereditary pancreatitis	KLK12
5406	PNLIP	Carboxylic ester hydrolase activity and triglyceride lipase activity	Pancreatic colipase deficiency and pancreatic lipase deficiency	LPL
1357	CPA1	Metallocarboxypeptidase activity and exopeptidase activity	Borna disease and pancreatitis, hereditary	CPA3
1360	CPB1	Metallocarboxypeptidase activity and carboxypeptidase activity	Acute pancreatitis and tricuspid valve insufficiency	CPA3
63036	CELA2A	Serine-type endopeptidase activity and serine hydrolase activity	Pancreatitis, hereditary	CELA2B
5967	REG1A	Carbohydrate binding and growth factor activity	Acinar cell carcinoma and tropical calcific pancreatitis	REG3G
1056	CEL	Hydrolase activity and carboxylic ester hydrolase activity	Maturity-onset diabetes of the young, Type VIII and maturity-onset diabetes of the young	CES2

**Table 6:** The Acc and highest relevance score of these methods.

Dataset	*L*_1/2_ gLPCA		RgLPCA		gLPCA		*L*_0_ PCA		*L*_1_ PCA		PCA		LE	
	Acc (%)	Relevance score	Acc (%)	Relevance score	Acc (%)	Relevance score	Acc (%)	Relevance score	Acc (%)	Relevance score	Acc (%)	Relevance score	Acc (%)	Relevance score
AMLALL	**51.33**	**55.37**	49.88	46.11	48.67	46.11	40.00	38.15	52.00	46.11	49.00	46.11	49.60	46.11
PAAD-GE	**61.60**	**85.56**	60.51	61.01	59.40	61.01	43.80	54.77	47.20	54.77	57.20	82.20	61.40	82.20
